# Transporter oligomerization: form and function

**DOI:** 10.1042/BST20160217

**Published:** 2016-12-02

**Authors:** Yilmaz Alguel, Alexander D. Cameron, George Diallinas, Bernadette Byrne

**Affiliations:** 1 Department of Life Sciences, Imperial College London, London SW7 2AZ, U.K.; 2 School of Life Sciences, University of Warwick, Gibbet Hill Road, Coventry CV4 7AL, U.K.; 3 Department of Biology, National and Kapodistrian University of Athens, Panepistimioupolis 15781 Athens, Greece

**Keywords:** integral membrane transporter, oligomerization, regulation of transporter function, trafficking, transporter function

## Abstract

Transporters are integral membrane proteins with central roles in the efficient movement of molecules across biological membranes. Many transporters exist as oligomers in the membrane. Depending on the individual transport protein, oligomerization can have roles in membrane trafficking, function, regulation and turnover. For example, our recent studies on UapA, a nucleobase ascorbate transporter, from *Aspergillus nidulans*, have revealed both that dimerization of this protein is essential for correct trafficking to the membrane and the structural basis of how one UapA protomer can affect the function of the closely associated adjacent protomer. Here, we review the roles of oligomerization in many particularly well-studied transporters and transporter families.

## Introduction

The formation of transporter oligomers has been studied by a range of methods, including fluorescence resonance energy transfer (FRET) [1], cross-linking [2], pull-downs [3] and co-immunoprecipitation [4], as well as biophysical characterization using, for example, size-exclusion chromatography–multiangle light scattering (SEC–MALS) [5]. While such approaches have effectively shown the formation of transporter oligomers, more recent investigations using dominant mutants and high-resolution structural studies have started revealing insights into the precise roles of oligomerization for transporter trafficking, function and regulation of function. This review uses many particularly well-studied transporters and transporter families to provide a brief overview of current understanding of the roles of transporter oligomerization. There are additional examples where high-resolution structures have revealed that the interface between transporter protomers in an oligomeric arrangement forms the substrate-binding site and translocation channel. Examples include the small multidrug transporter, EmrE [6] and ABC transporters [7,8]. In these cases, oligomerization is responsible for generating the correct architecture for both substrate binding and transport. These transporters are not covered in this review. Additionally, we have not included transporters where the sole purpose of oligomerization seems to be for stability.

## The nucleobase ascorbate transporters

In eukaryotes, transporters are assembled in the endoplasmic reticulum (ER) before being trafficked through COPII vesicles [9]*.* For some membrane proteins, the association of protomers into the correct quaternary arrangement seems to form a key quality control in this trafficking process [10]. This also seems to be true for the H^+^ uric acid–xanthine symporter, UapA, a nucleobase ascorbate transporter (NAT) from *Aspergillus nidulans*, which requires correct association of individual protomers into a dimer for effective exit from the ER and localization to the plasma membrane [5]. Mutations in TM7 appear to inhibit or impair oligomerization, preventing efficient trafficking to the membrane and increased turnover. The recent structure of UapA (see below) revealed that TM7 is not directly involved in the dimer interface, but structural changes in this region may indirectly inhibit dimer formation. The importance of oligomerization for correct trafficking to the plasma membrane has been reported for a range of other transporters, notably the neurotransmitter sodium symporter family (see below). However, it remains unclear precisely why oligomers need to form in the ER. The most plausible explanation is that the oligomeric arrangement requires the protomers to be correctly folded, with oligomer formation likely to act as a quality control, allowing only proteins in the fully folded state to be trafficked. Interestingly, in the case of UapA, impaired trafficking can be overcome by co-expression of the TM7 mutants with wild-type (WT) UapA [5], suggesting, at least in this context, a dominant positive effect of the WT form.

**Figure 1.**
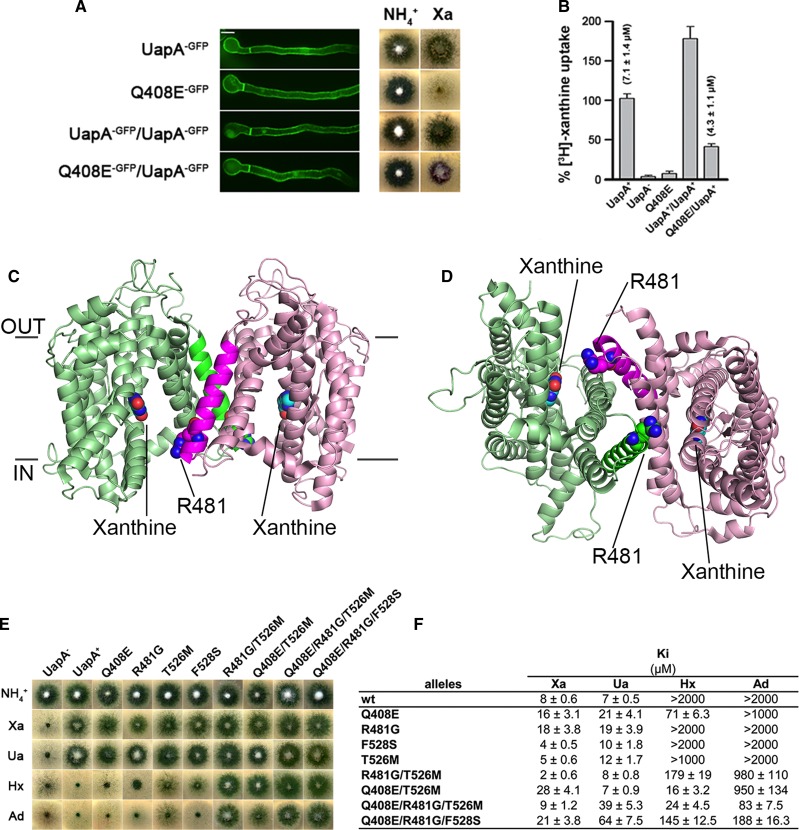
Role of oligomerization of UapA. (**A**) Co-expression of WT UapA and a non-functional mutant Q408E in a UapA knockout strain of *A. nidulans*. Both forms traffic effectively to the membrane as indicated by GFP fluorescence, but the fungi only grow effectively in the presence of xanthine when expressing from one or two copies of the WT *uapa* gene. (**B**) Comparison of xanthine uptake in *A. nidulans* cells expressing the WT and mutant forms of UapA. UapA^−^ indicates the UapA knockout strain. (**C**) Structure of the dimer of UapA from *A. nidulans* (PDB: 5I6C) shown looking through the membrane and (**D**) from the intracellular side of the protein. In each case, one protomer is shown in light green and one in light pink with TM13 shown in bright green and magenta, respectively. Xanthine is shown in cyan space-filling model, and R481 is shown in bright green and magenta space-filling model. For clarity, TMs 6 and 7 have been removed. (**E**) Growth of the UapA^−^ strain expressing WT or substrate selectivity mutant UapA on a range of native (uric acid or xanthine) or non-native (hypoxanthine or adenine) substrates. (**F**) Transport characteristics of these mutant proteins. Note that the R481G mutant allows transport of non-native substrates.

In addition, dominant negative mutants of UapA have been described, which traffic effectively to the membrane but reduce the transport activity of co-expressed WT, strongly suggesting that this transporter also functions as a dimer (Figure 1a,b) [11]. The high-resolution structure of a thermostabilized form of UapA, trapped in the inward-facing conformation, has revealed further clues about the role that oligomerization has in transport function [11]. UapA was crystallized as a closely associated dimer (Figure 1c,d), confirming the earlier findings [5]. There are many key residues in UapA involved in substrate selectivity [12–14]. The structure revealed that one of these residues, Arg481, lies in close proximity to the binding site of the opposite protomer, and it is likely to act as the last checkpoint, allowing efficient uptake of the native substrates, xanthine and uric acid, and not other related molecules. The size of the side chain is likely to be important here as mutation to the much smaller Gly allows uptake of adenine and hypoxanthine, substrates not transported by WT UapA (Figure 1e,f) [11]. While the structure highlighted the interdependency of the UapA protomers in substrate selectivity, it did not explain the dominant negative effects seen for the Q408E mutant. Thus, there are clearly other aspects of UapA protomer cross-talk that have yet to be elucidated. It is interesting to note that, in most crystal structures of dimeric transporters, the individual protomers adopt similar conformations. One exception to this is the recent structure of the CitS where an asymmetrical dimer is observed [15]. In this case, both outward- and inward-facing conformations can be seen in a single oligomer. While such arrangements appear to be stochastic, it is intriguing to ask whether such arrangements may be a co-ordinated part of the transport cycle. The UapA construct used for structure determination is a conformationally locked mutant; however, it is possible that, in the WT form, the two protomers can adopt different conformational states as seen in CitS. To date, there is no evidence that the CitS dimer is important for function.

The overall structure of UapA is similar to that of the anion exchanger 1 (AE1) [16]. Previous research had indicated that AE1 formed dimers [17], and this was confirmed in the high-resolution structure. Intriguingly, although it seems likely that UapA and AE1 function using a very similar mechanism, dimer formation is markedly different in the two proteins. In the case of UapA, the buried interface is ∼6000 Å^2^, and it involves mainly interactions between TMs 12, 13 and the loop between the two TMs with TM13, particularly closed associated with the opposite protomer [11]. In contrast, the buried interface in the AE1 dimer is substantially smaller at ∼1100 Å^2^, and only the extracellular tip of TM6 and the loop regions between TMs 5 and 6, 6 and 7 and 12 and 13 are involved in dimer formation [16]. It should be noted, however, that the same subdomain, the so-called gate domain, is involved in dimerization in both proteins. There are no data so far to support a role for dimerization in the function of AE1. It seems likely that the structural and functional differences of oligomerization observed are protein specific, even for transporters that operate in a very similar way. The UapA and AE1 proteins are in the inward- and outward-facing conformations, respectively, which may account for some of the observed differences. It should be noted that in the case of the sodium–proton antiporter, NapA, the dimer interface is very similar in the crystal structures of both the inward- and outward-facing conformations [18,19], although it is not clear whether dimerization is important for function in this protein. It is still possible though that the variation between the UapA and AE1 dimer interfaces is the result of differences in the crystallization conditions used in each case and/or the fact that the AE1 structure lacks the large soluble N-terminal region.

The formation of the UapA dimer is largely mediated by hydrophobic interactions involving residues that are not particularly well conserved among NATs. The structure of the bacterial NAT, UraA, was described as a monomer, but analysis of the crystal contacts indicates that there are interactions between protomers. These result in the formation of a loose UraA dimer and involve similar regions of the protein as seen in the dimer interface of UapA and AE1 [20]. The interaction interface of UraA is much smaller than that of UapA, involving only a handful of residues from TM13 and the loop between TMs 12 and 13 [20]. The functional significance of dimerization of UraA, or indeed other members of the NAT family, remains to be determined.

## The LeuT superfamily

The role of oligomerization of neurotransmitter sodium symporter (NSS) family transporters has been particularly intensively studied. Oligomerization has been shown to be important for effective membrane trafficking of the glycine transporter, GlyT2 [3], and the GABA transporter 1, GAT-1 [21]. There are also many studies revealing the key role of oligomerization in direct transporter function. Dominant negative mutants of the human dopamine transporter (DAT) have been shown to reduce the function of co-expressed WT transporters, without any effect on trafficking to the plasma membrane [22]. Further functional studies have supported co-operativity, or cross-talk, between individual protomers in DAT oligomers [23]. Additional supportive evidence for this cross-talk has come from studies on fusion proteins of the serotonin transporter (SERT) with GAT-1 [24]. Passage of serotonin through SERT in the heteroligomer arrangement inhibits transport of GABA through GAT-1. Furthermore, the transport of amphetamine analogues, specific only to SERT, increases GABA export [24]. These studies are strongly suggestive that the activity of one protomer can influence the activity of the associated molecule(s).

There is no clear structural motif associated with NSS transporter oligomerization. Leucine zipper motifs located in TM2 have been suggested as key in oligomerization of both DAT [22] and GAT-1 [21]. Several studies indicate the importance of the GXXXG motifs in dimerization of integral membrane proteins where the two Gly residues located on the same side of a transmembrane α-helix allow tight packing of protomers [25,26], and such a motif exists in TM6 of the NSS transporters. An additional oligomer interaction site has been suggested in TMs 11 and 12 of SERT [27].

It is interesting that although there is substantial evidence for oligomerization of both DAT and SERT [28], the crystal structures, to date, have failed to capture the oligomeric forms [29–31]. This might be due to the formation of relatively weakly associated oligomers unable to withstand the rigours of the extraction and isolation processes required for structural studies. This issue has also been highlighted for the recent structure of the monomeric form of an SLC26 family member, where loss of the oligomeric form was assumed to be a result of the harsh nature of the detergent treatment [32]. It remains to be seen if oligomerization is important for the function of SLC26. It is also worth noting that all the DAT and SERT structures available have been obtained in complex with antibody fragments. Antibody–transporter complex formation may disrupt oligomer interactions. Perhaps, approaches using fusion proteins of the individual protomers might facilitate trapping the oligomeric state of the NSS transporters.

**Figure 2.**
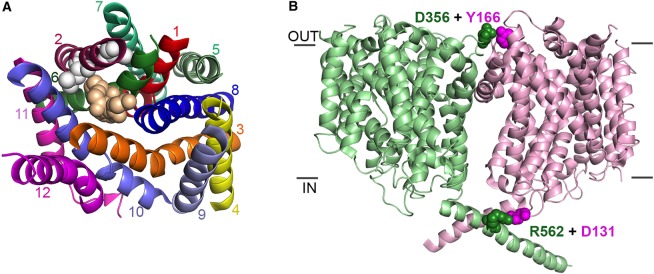
Monomer versus oligomer transporter structures. (**A**) Structure of the dopamine transporter from *Drosophila melanogaster* (PDB: 4M48). For clarity, only the transmembrane regions are shown. Each transmembrane region is individually coloured and labelled. The residues of the predicted leucine zipper motif and the GXXXG motif are shown in white and wheat coloured space-filling model, respectively. (**B**) Key ionic interactions between protomers of the BetP trimer from *Corynebacterium glutamicum* (PDB: 3P03). Only two of the protomers are shown for clarity, one in light green and one in light pink. The residues involved are coloured according to the protomer, and are shown in space-filling representation.

The monomeric structures of DAT [29,30] and SERT [31] reveal that both the Leu zipper motif and the GXXXG motif are buried away within the core of the protein, more likely to have roles in the architecture of the protomer than in mediating oligomerization (Figure 2a). Mutations in either of these regions may distort the protomer so much, that it can no longer form effective oligomers. Both TMs 11 and 12 are surface exposed, and they may have roles in oligomer formation (Figure 2a). Indeed, in the structure of SERT, TM12 is involved in the formation of a crystallographic dimer [31]. This is highly unlikely to represent a physiological arrangement of the molecules, however, as one protomer is rotated substantially compared with the other. This would put the soluble loop regions of one of the protomers into the membrane, a highly unlikely arrangement. TM12 may have roles in interactions with lipids as, in the structure, this region of the protein is associated with cholesterol hemisuccinate [31].

Dimer structures of the bacterial NSS homologue, LeuT, are available however, and these reveal that dimerization is mediated by TM12 and TM9a as well as residues from the second extracellular loop [33]. However, there is no evidence as yet that LeuT functions as an oligomer.

Studies on the bacterial osmoregulated betaine transporter BetP, a member of the betaine/choline/carnitine family, have shown that this protein forms a stable trimer [34]. Generation of mutants of the transporter reveals that the protein is still transport-active as an individual protomer, although the activity is reduced compared with that of the WT trimer [35]. However, the monomeric form is no longer responsive to osmotic stress, strongly suggesting that the trimer is key in detecting environmental change and activating the transporter in response to that change [35]. Recent structures have additionally highlighted that the interactions involved in trimer formation may play roles in the conformational changes associated with the transport cycle [36]. Both functions of the BetP trimer appear to be regulated by ionic interactions formed between the individual protomers [37] (Figure 2b). Intriguingly, as seen in CitS, the individual protomers in the BetP trimer can adopt different conformations [36].

## The SWEET transporter family

SWEET transporters are responsible for uptake and distribution of mono- and disaccharides. Early studies on plant SWEET transporters using dominant negative mutants highlighted the importance of oligomerization for function [38]. These transporter proteins contain just seven transmembrane domains, and so it was thought that oligomerization was essential to form the functional translocating unit. However, the recent structure of the homotrimeric SWEET from *Arabidopsis thaliana* shows that the individual protomer forms the translocation channel [39]. Further structure-based investigations revealed that functionally inactive mutations in the extracellular or intracellular gates of one protomer negatively affected the function of associated protomers [39]. It is possible that the lack of conformational change in one protomer inhibits movement in associated molecules. These findings strongly suggest that SWEET homotrimers function through co-operativity between protomers; however, the precise molecular basis of this is yet to be revealed.

## The major facilitator superfamily

Early studies on the bacterial major facilitator superfamily (MFS) transporter, LacS, gave the first indications that oligomerization may play a role in transporter function. Biophysical analyses had indicated that LacS was a dimer [40]. Co-expression of mutant non-functional transporter with functional transporter forms indicated co-operativity between the individual protomers of the dimer required for proton-dependent transport [41].

A dominant positive mutant has been described for the human proton-coupled folate transporter. Co-expression of the WT transporter with the mutant, which traffics effectively to the membrane but is transport-inactive, resulted in greater levels of activity than expression of WT alone [1]. Indeed, the level of activity observed was similar to that for co-expression of two differently tagged forms of WT transporter. This indicated a dominant positive effect whereby the active WT was able to recover the transport activity of the inactive mutant through direct association of the different transporter protomers [1].

Research on the plant phosphate transporter, Pht1, another MFS member, identified a mutant with expression levels and localization similar to WT, but which both reduced oligomerization and increased transport activity (*V*_max_). These findings strongly suggested that, in this case, oligomerization plays a regulatory role [42].

**Figure 3.**
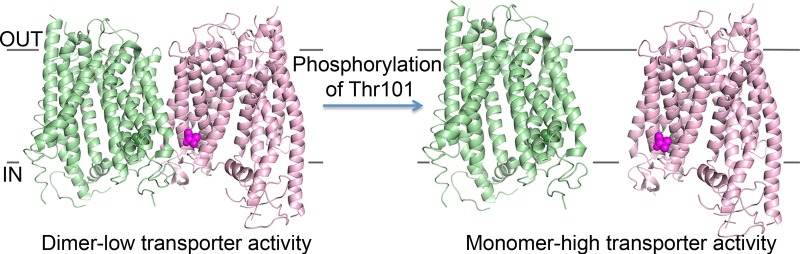
Phosphorylation-dependent regulation of NRT1.1 from *Arabidopsis thaliana* (PDB: 4OH3). In the dimer form, T101, shown in space-filling representation, is unphosphorylated, and the protein functions as a low-affinity transporter. Upon phosphorylation, the dimer dissociates and the individual protomers function independently as high-affinity transporters.

Oligomerization-dependent regulation has also been reported for the plant nitrate transporter, NRT1.1, which can act as either a low- or high-affinity transport system [43]. Crystal structures of the NRT1.1 revealed a dimeric form of the protein (Figure 3) [44,45]. Mutational studies indicated that phosphorylation of Thr101 resulted in reduced stability and increased transport activity compared with the unphosphorylated form [44]. Sun et al. [45] suggested that this was due to a change in the oligomeric status of the protein, and they carried out experiments, indicating that phosphorylation of Thr101 induced dissociation of the low-affinity dimer into high-affinity protomers (Figure 3). However, another plausible theory is that phosphorylation simply results in a minor structural change with the effect of changing the affinity [44]. Support for this theory came from the generation of a phosphomimic mutant of the equivalent residue in the related monomeric peptide transporter from *Shewanella oneidensis*, PepT_so_, which also decreased stability and increased transport activity [44].

## The ammonium transporter/MEP/Rh transporter family

The plant ammonium transporters (AMTs), members of the AMT/MEP/Rh transporter family, are also regulated by phosphorylation. In contrast with NRT1.1, phosphorylation of C-terminal residues in AMTs [46] leads to rapid transporter inactivation. Recent evidence has shown that these proteins can exist as either homo- or heterotrimers. Co-expression of WT and a mutant containing a phosphorylation mimic amino acid (Thr to Asp substitution) reduces ammonium uptake in a dominant negative manner, showing that phosphorylation of one protomer inhibits the activity of associated non-phosphorylated protomers [47,48]. Such regulatory mechanisms in the Pht1, NRT1.1 and the AMTs are likely to allow plants to rapidly and effectively adapt to changing environmental conditions.

## Conclusion

It is clear that oligomerization is key to correct localization, function and regulation of different transporter proteins. Combinations of functional and structural studies have been particularly effective at providing insights into the molecular basis of functional cross-talk of transporter protomers in oligomeric arrangements. However, it is clear that we have only just scratched the surface of the precise roles of this important feature of many biologically and medically important transporter proteins.

## Abbreviations

AE1: anion exchanger 1
AMTs: ammonium transporters
DAT: dopamine transporter
ER: endoplasmic reticulum
FRET: fluoresence resonance energy transfer
GAT1: GABA transporter 1
MFS: major facilitator superfamily
NATs: nucleobase ascorbate transporters
NSS: neurotransmitter sodium symporter
SERT: serotonin transporter
WT: wild type
